# Decomposing the educational inequalities in the factors associated with severe acute malnutrition among under-five children in low- and middle-income countries

**DOI:** 10.1186/s12889-020-08635-3

**Published:** 2020-04-25

**Authors:** A. F. Fagbamigbe, N. B. Kandala, O. A. Uthman

**Affiliations:** 1 Department of Epidemiology and Medical Statistics, Faculty of Public Health, College of Medicine, University of Ibadan, Ibadan, Nigeria; 2 Division of Health Sciences, Populations, Evidence and Technologies Group, Warwick Medical School, University of Warwick, Coventry, UK; 3 Department of Mathematics, Physics & Electrical Engineering, Northumbria University, Newcastle upon Tyne, UK

**Keywords:** Severe acute malnutrition, LMIC, Educational inequalities, Blinder-Oaxaca decomposition

## Abstract

**Background:**

Low- and Middle-Income Countries (LMIC) have remained plagued with the burden of severe acute malnutrition (SAM). The decomposition of the educational inequalities in SAM across individual, household and neighbourhood characteristics in LMIC has not been explored. This study aims to decompose educational-related inequalities in the development of SAM among under-five children in LMIC and identify the risk factors that contribute to the inequalities.

**Methods:**

We pooled successive secondary data from the Demographic and Health Survey conducted between 2010 and 2018 in 51 LMIC. We analysed data of 532,680 under-five children nested within 55,823 neighbourhoods. Severe acute malnutrition was the outcome variable while the literacy status of mothers was the main exposure variable. The explanatory variables cut across the individual-, household- and neighbourhood-level factors of the mother-child pair. Oaxaca-Blinder decomposition method was used at *p* = 0.05.

**Results:**

The proportion of children whose mothers were not educated ranged from 0.1% in Armenia and Kyrgyz Republic to as much as 86.1% in Niger. The overall prevalence of SAM in the group of children whose mothers had no education was 5.8% compared with 4.2% among those whose mothers were educated, this varied within each country. Fourteen countries (Cameroon(*p* < 0.001), Chad(*p* < 0.001), Comoro(*p* = 0.047), Burkina Faso(*p* < 0.001), Ethiopia(*p* < 0.001), India(*p* < 0.001), Kenya(*p* < 0.001), Mozambique(*p* = 0.012), Namibia(*p* = 0.001), Nigeria(*p* < 0.001), Pakistan(*p* < 0.001), Senegal(*p* = 0.003), Togo(*p* = 0.013), and Timor Leste(*p* < 0.001) had statistically significant pro-illiterate inequality while no country showed statistically significant pro-literate inequality. We found significant differences in SAM prevalence across child’s age (*p* < 0.001), child’s sex(*p* < 0.001), maternal age(*p* = 0.001), household wealth quintile(*p* = 0.001), mother’s access to media(*p* = 0.001), birth weight(*p* < 0.001) and neighbourhood socioeconomic status disadvantage(*p* < 0.001). On the average, neighbourhood socioeconomic status disadvantage, location of residence were the most important factors in most countries. Other contributors to the explanation of educational inequalities are birth weight, maternal age and toilet type.

**Conclusions:**

SAM is prevalent in most LMIC with wide educational inequalities explained by individual, household and community-level factors. Promotion of women education should be strengthened as better education among women will close the gaps and reduce the burden of SAM generally. We recommend further studies of other determinate causes of inequalities in severe acute malnutrition in LMIC.

## Background

Malnutrition among under-five children (U5C) remains both a social and public health burden [1, 2] especially in the Low- and Middle-Income Countries (LMIC). The World Health Organisation (WHO) maintains that malnutrition is responsible, directly or indirectly, for 35% of deaths among U5C [3], among which is Severe Acute Malnutrition (SAM). SAM is the most extreme and visible form of undernutrition among U5C. Under-five children with SAM usually “have very low weight for their height and severe muscle wasting” [4]. The likelihood that a child with SAM will eventually die is very high [4, 5]. Besides, “children with severe acute malnutrition are nine times more likely to die than well-nourished children” [4]. The UNICEF (United Nations International Children’s Emergency Fund) reported that SAM affected more than 16 million children globally in 2016 [4]. Although this figure is staggering, it is likely to have been underestimated [5].

To reduce the burden of SAM, there is a need to implement multi-sectoral evidence-based interventions which will enhance child and maternal health [3] in the long run. However, the development of the appropriate strategies, programmes and policies on the reduction of SAM, is hinged on the availability of information that can enhance child health interventions. While the literature is replete with the factors predisposing children to SAM and other poor nutrition outcomes, decomposition of these factors on key variables significant to poor nutrition is scarce in the literature. The identified factors are largely individual and household factors such as food insecurity, inadequate care and feeding, unhealthy environment, poor access to education, child’s age and sex, and mothers’ employment status and income [1, 6–12]. In a recent hierarchical analysis of factors associated with SAM in 51 LMIC, Fagbamigbe et al. identified maternal educational attainment, household wealth status and rural-urban differentials in the location of residence as the main determinants of whether a child has SAM or not [13]. These findings motivated the current study.

There is a paucity of data on SAM in LMIC, especially on its decomposition by maternal educational differences, which has limited the understanding of the magnitude of the challenges of SAM for fact-based interventions. This is despite UNICEF’s recommendation that complex social and political efforts are crucial to ending SAM [4]. The role(s) of educational inequalities in the distribution of SAM in the LMIC and factors associated with the inequalities have not received sufficient attention. A recent Ghanaian study found a high level of influence of educational inequalities on all factors associated with malnutrition in the study [14]. Amongst others, the study showed that the nutritional status of children from educated mothers are generally better than among those from uneducated mothers and some factors influence these differentials.

Inequalities in maternal education remain key barriers to the occurrence of SAM among U5C [9, 11, 12, 15–18]. However, the underlying causes of educational inequalities in the development of SAM among U5C remain poorly operationalized, studied and understood. There is, therefore, a need to understand what influences the wide gap in the development of SAM among children from educated and uneducated mothers. In an attempt to understand the factors that explain the educational-related inequalities in the development of SAM among U5C in the LMIC and propose necessary strategies for interventions, we assessed the level of educational inequalities in LMIC and examined the factors associated with these inequalities in the development of SAM among U5C in LMIC. We were motivated to identify the causes and the extent of the variabilities of the educational-related inequalities in the development of SAM among U5C in the LMIC beyond compositional characteristics. A good understanding of the gaps in the development of SAM among U5C in the LMIC would guide interventions for improving child nutrition.

## Methods

### Study design and data

The nationally representative cross-sectional data obtained from successive Demographic and Health Surveys (DHS) conducted in LMIC was used for this study. We extracted data from 51 most recent successive DHS conducted between 2010 and 2018 which were available as of March 2019 and these included under-five children (U5C) anthropometry data. Typically, the DHS uses stratified multi-stage sample drawing techniques with clusters (neighbourhoods) as the primary sampling unit (PSU) [19, 20]. Country-specific sampling methodologies are also available at dhsprogram.com and in report forms [21–23]. Within each sampled household, individuals that meet the inclusion criteria were interviewed. Sampling weights were calculated to adjust for unequal probabilities of selection including non-responses. Application of sample weights and adjustment for non-responses make the findings from the surveys to fully represent the target populations. All the DHS questionnaires were standardized and implemented across the various countries using similar training of the interviewers, supervision of the interviewers as well as the implementation protocols. In this study, we used the DHS children recode data. The data covered the health experiences of under-five children born to sampled women within five years preceding the survey date. The anthropometry measurements were taken using standard procedures.

### Dependent variable

The dependent variable in this study is severe acute malnutrition defined as “a very low weight for height score (WHZ) below -3 z-scores of the median WHO growth standards, by visible severe wasting, or by the presence of nutritional oedema” [3]. The z-scores are composite scores computed using the weight and height of the children. We generated z-scores using the WHO-approved methodologies [24] and categorized children with z-scores <− 3 standard deviation as having SAM (Yes = 1) and as No = 0 if otherwise.

### Main determinant variable

Maternal education was used as a proxy for literacy in this study. Literacy is a key skill and an important measure of a population’s level of education. Literacy is the ability to both read and write a short, simple statement about one’s own life [25]. We, therefore, categorized education as having no formal education (Illiterate) and educated (can read and write: have a minimum of completed primary education - Literate).

### Independent variables

#### Individual-level factors

These include sex of the children (male or female), children age (under 1 year and 12–59 months), maternal age (15–24, 25--34, 35–49 years), occupation (currently working or not), access to media (at least one of radio, television, or newspaper), sources of drinking water (improved or unimproved), toilet type (improved or unimproved), weight at birth (average+, small, and very small), ability to pay for health care, health insurance coverage, birth interval (firstborn, < 36 months, and > 36 months) and birth order (1, 2, 3, and 4+). We used the DHS-generated wealth index as an alternative indicator for the households’ Socio-Economic Status (SES). The methods used in the computation of the DHS wealth index have been published previously [26].

#### Neighbourhood-level factors

In this study, the term “neighbourhood” was used to depict people living in the same cluster within the same geographical setting. The neighbourhoods were mapped out to include households of the same clusters otherwise referred to as sharing the same PSU across each of the countries studied [19, 20]. Operationally, we defined “neighbourhood” as clusters and “neighbours” as a member of the same cluster. The PSUs were identified using the most recent census in each country where DHS was carried out. Among the community-level variables generated is the neighbourhood socioeconomic disadvantage. It was generated using principal component analysis of the proportion of respondents living in rural areas, with no education, unemployed, and belonging to the lowest two wealth quintiles.

### Statistical analyses

In this study, we carried out analytical analyses comprising descriptive statistics and multivariate analysis. Univariable and bivariable analysis were used to describe the study population. Descriptive statistics was used to depict the distribution of respondents by country and the explanatory variables. Estimates of the frequencies were expressed as percentages and confidence intervals. Secondly, we computed the risk difference in the development of SAM between U5C whose mothers were literate and the others that were not literate. A risk difference (RD) greater than (RD > 0) suggests that SAM is more prevalent among children born to mothers with no formal education (pro-illiterate inequality). Conversely, an RD < 0 indicates that SAM is more prevalent among children whose mothers were educated (pro-educated inequality).

Thirdly, in the multivariabe analysis, the logistic regression analysis using the pooled data from the 51 LMIC was used to carry out a Blinder-Oaxaca decomposition analysis (BODA) [27, 28]. The Blinder-Oaxaca decomposition assumes that children whose mothers were uneducated share the same characteristics with the children of educated mothers. Our choice of the Blinder-Oaxaca method is hinged on the fact that it allows for the decomposition of the differentials in the determinate variable between the two groups of the children into two components so that the gaps can be seen more clearly [29–31]. The first component of the decomposition is the “explained” portion (also known as the “compositional” or “endowments”) of the gap that shows the differentials in the distributions of the quantifiable characteristics of interest among these groups. The BODA method enabled the quantification of the magnitude of the gap between “the advantaged” and “the disadvantaged” groups is attributable to differentials in the specific quantifiable characteristics. The second component of the decomposition analysis is the “unexplained” part (also referred to as the structural component) which shows the magnitude of the gap caused by the differentials in the regression coefficients and the unmeasured characteristics between these two groups of children being compared. The methods used in the current study have been used in previous and related studies [13, 29–31].

## Results

### Sample characteristics

We analysed data of 532,680 under-five children nested within 55,823 neighbourhoods from 51 LMIC who participated in the DHS between 2010 and 2018. The regions of the world, countries, year of data collection, numbers of neighbourhoods, number of under-five children, percentage of the uneducated mothers and the weighted prevalence of SAM among children of uneducated and educated mothers are listed in Table 1. The proportions of children whose mothers had no formal education ranged from 0.1% in Armenia and Kyrgyz Republic to 86.1% in Niger and a median of 20.1% in Haiti.

**Table 1:** Description of Demographic and Health Surveys data by countries and SAM prevalence among under-five children in LMIC, 2010–2018

Country	Year of Survey	Number of Under-5 Children	Weighted SAM prevalence (%)	Weighted Uneducated (%)	*Weighted SAM (%) Uneducated	Weighted SAM (%) Educated
All		532,680	4.7	31.1	5.8	4.2
Eastern Africa		67,418	1.5	29.4	2.5	1.1
Burundi	2016	6052	0.9	47.5	0.9	0.9
Comoro	2012	2387	3.9	47.8	*4.9	*2.9
Ethiopia	2016	8919	3.0	65.8	*3.5	*2.0
Kenya	2014	18,656	1.0	11.9	*2.3	*0.8
Malawi	2016	5178	0.6	13.3	0.5	0.6
Mozambique	2011	9313	2.1	37.6	*2.6	*1.9
Rwanda	2015	3538	0.6	14.4	0.9	0.6
Tanzania	2016	8962	1.3	21.5	1.5	1.2
Uganda	2016	4413	1.4	11.2	2.0	1.3
Middle Africa		37,136	2.5	32.4	4.1	1.8
Angola	2016	6407	1.0	28.9	1.4	0.9
Cameroon	2010	5033	1.9	26.2	*4.3	*1.0
Chad	2015	9826	4.3	65.3	*5.2	*2.3
Congo	2012	4475	1.6	7.0	2.8	1.5
DRC	2014	8059	2.7	19.3	2.7	2.7
Gabon	2012	3336	1.2	6.9	1.6	1.1
Northern Africa		13,682	3.8	17.9	4.3	3.7
Egypt	2014	13,682	3.8	17.9	4.3	3.7
Southern Africa		20,273	1.7	7.2	2.3	1.6
Lesotho	2016	1312	0.7	0.9	0.0	0.7
Namibia	2013	1558	2.2	6.8	*7.9	*1.7
South Africa	2016	1082	0.5	2.1	3.1	0.5
Zambia	2014	11,407	2.1	11.2	2.0	2.1
Zimbabwe	2015	4914	1.1	1.2	0.0	1.1
Western Africa		85,462	4.7	60.8	5.4	3.7
Benin	2018	12,033	1.1	65.7	1.2	0.9
Burkina Faso	2010	6532	5.8	83.8	6.1	4.5
Cote d’Ivoire	2012	3200	1.8	64.8	1.7	2.0
Gambia	2013	3098	4.7	59.6	4.9	4.4
Ghana	2014	2720	0.7	28.8	0.9	0.7
Guinea	2012	3085	3.7	78.7	4.1	2.4
Liberia	2013	3171	2.2	42.5	2.1	2.3 4.5
Mali	2013	4306	5.1	82.9	5.2	4.5
Niger	2012	4771	6.2	86.1	6.2	6.2
Nigeria	2013	24,505	8.8	46.4	*11.9	*6.2
Senegal	2017	10,787	1.5	61.6	*1.9	*1.0
Sierra Leone	2013	4069	3.8	69.8	3.6	4.3
Togo	2014	3185	1.6	40.6	*2.2	*1.1
Central Asia		9883	1.5	1.7	1.0	1.6
Kyrgyz Republic	2012	4016	1.1	0.1	0.0	1.1
Tajikistan	2017	5867	1.8	2.7	1.0	1.8
South-Eastern Asia		4324	2.4	13.2	2.9	2.4
Cambodia	2014	4324	2.4	13.2	2.9	2.4
Southern Asia		240,849	7.1	29.4	7.8	6.8
Bangladesh	2014	6965	3.1	16.3	3.0	3.1
India	2016	225,002	7.4	29.7	*8.1	*7.1
Maldives	2016	2362	2.0	1.2	0.0	2.0
Nepal	2016	2369	1.9	34.5	1.7	2.0
Pakistan	2018	4151	2.3	48.6	*2.6	*2.1
Western Asia		1561	1.5	0.1	0.0	1.5
Armenia	2016	1561	1.5	0.1	0.0	1.5
Central America		21,717	0.2	12.6	0.1	0.2
Guatemala	2012	11,744	0.1	18.6	0.0	0.1
Honduras	2016	9973	0.3	4.9	0.4	0.3
South America		9213	0.1	3.1	0.3	0.1
Peru	2012	9213	0.1	3.1	0.3	0.1
South Europe		2462	0.5	1.1	2.7	0.5
Albania	2018	2462	0.5	1.1	2.7	0.5
Caribbean		18,700	3.9	17.7	6.7	3.3
Dominica	2013	3187	0.6	2.2	1.2	0.6
Haiti	2016	5598	0.9	20.1	1.2	0.8
Myanmar	2016	4197	1.4	16.6	1.4	1.4
Timor-Leste	2016	5718	9.9	24.4	*13.4	*8.8

*Significant at 0.05 in Mantel Haenszel test of homogeneity of the odds ratio

### Prevalence of SAM by countries and maternal education

**Fig. 1:**
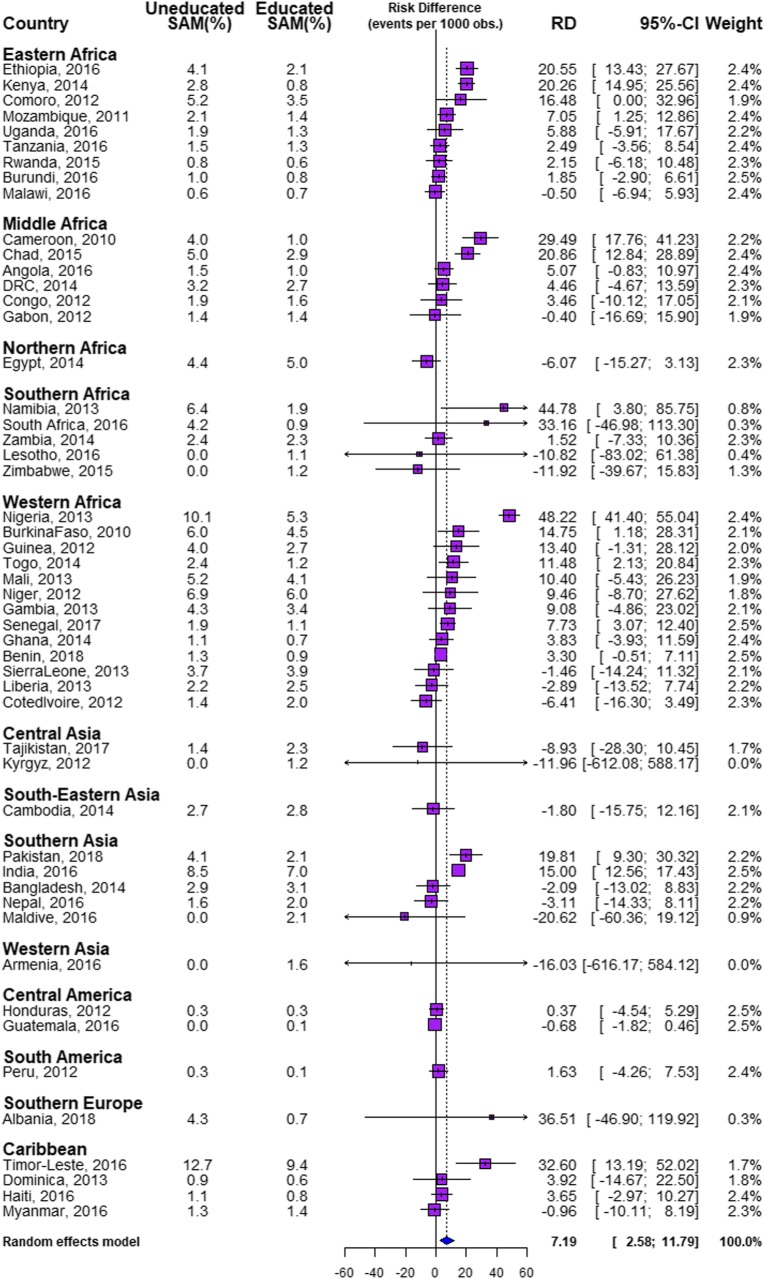
Risk difference between children from uneducated and educated mothers in the prevalence of SAM by countries

We found differences in the prevalence of SAM among children of educated and uneducated mothers in the 51 LMIC studied (Table 1 and Fig. 1). The overall SAM prevalence was 4.7% with a median prevalence of 1.8% ranging from 0.1% in Guatemala to 9.9% in Timor-Leste as shown in Table 1. The prevalence of SAM among children of uneducated mothers ranged from 0.0% in Lesotho, Zimbabwe, Kyrgyz Republic , Armenia and Guatemala to 12.7% in Timor-Leste, while the prevalence ranged from 0.1% in Peru and Guatemala to 9.4% in Timor-Leste among children of the educated mothers. We used the Mantel Haenszel test of homogeneity of odds ratio to test the statistical significance of the association between the explanatory variables with literacy level as an effect modifier. We found significant pro-illiterate inequalities in fourteen countries: Cameroon (*p* < 0.001), Chad (*p* < 0.001), Comoro (*p* = 0.047), Burkina Faso (*p* < 0.001), Ethiopia (*p* < 0.001), India (*p* < 0.001), Kenya (*p* < 0.001), Mozambique (*p* = 0.012), Namibia (*p* = 0.001), Nigeria (*p* < 0.001), Pakistan (*p* < 0.001), Senegal (*p* = 0.003), Togo (*p* = 0.013), and Timor Leste (*p* < 0.001) but no country has pro-educated inequalities as shown in Table 1.

### Prevalence of SAM by children characteristics and maternal education

In Table 2, we present the descriptive statistics of the characteristics of children across the 51 LMIC. About 51% of the children were males while only 20% were infants. About 53% of the children are from mothers were aged 25–34 years old while about 31% had no formal education. Nearly one-third of the mothers were unemployed at the time of data collection. The overall prevalence of SAM in the group of children whose mothers had no education was 5.8% compared with 4.2% among those whose mothers were educated. The Mantel Haenszel test of homogeneity of odds ratio showed that all the characteristics considered were independently significant. For instance child’s age (*p* < 0.001), child’s sex (*p* < 0.001), maternal age (*p* = 0.001), household wealth quintile (*p* = 0.001), mother’s access to media (*p* = 0.001), birth weight (*p* < 0.001) and neighbourhood socioeconomic status disadvantage (*p* < 0.001) had significant differences in SAM prevalence viz-a-viz mothers’ literacy (Table 2). Infants, male children and mothers at extreme age intervals; 15 to 24 and 34 to 49 had overall higher SAM prevalence. For wealth index, births of women from lowest wealth quintile had the highest rate of SAM within the “uneducated” group compared with those from richest wealth quintile (6.8% vs 3.4%) but the margins were closer within the “educated” group.

**Table 2:** Summary of pooled sample characteristics of the studied children in 51 LMIC

Characteristics	Weighted n	Weighted %	Weighted (%) Uneducated	Weighted SAM (%) Uneducated	Weighted SAM (%) Educated
Individual Level					
Age					
< 12 Months	103,379	20.0	29.0	*9.0	*6.7
12–59 Months	413,718	80.0	31.7	5.1	3.5
Sex					
Female	252,541	48.8	31.5	*5.4	*3.8
Male	264,556	51.2	30.8	6.3	4.5
Maternal Age					
15–24	160,133	31.0	22.4	*6.7	*4.8
25–34	273,802	52.9	31.8	5.8	4.1
35–49	83,162	16.1	45.7	5.1	2.7
Wealth Index					
Poorest	122,991	23.8	54.5	*6.8	*4.3
Poorer	112,755	21.8	37.0	5.7	4.4
Middle	104,194	20.1	26.4	5.3	4.2
Richer	96,896	18.7	18.3	4.4	4.2
Richest	80,261	15.5	8.8	3.4	3.8
Employment					
Yes	366,033	70.8	31.7	*5.9	*4.6
No	151,064	29.2	31.1	5.5	3.2
Access To Media					
No	188,357	36.5	55.8	*6.1	*4.3
Yes	328,311	63.5	17.0	5.3	4.1
Drinking-Water Sources					
Unimproved	95,544	19.2	43.9	*5.4	*3.1
Improved	402,688	80.8	28.7	5.9	4.3
Toilet Type					
Unimproved	248,331	49.9	45.3	*6.0	*4.4
Improved	249,753	50.1	18.1	5.2	3.9
Marital Status					
Never Married	12,199	2.4	10.0	*3.5	*1.7
Currently Married	484,949	93.8	32.0	5.9	4.3
Formerly Married	19,946	3.9	23.5	4.1	1.8
Weight At Birth					
Average+	423,017	85.4	30.4	*5.7	*4.2
Small	52,939	10.7	33.5	6.0	4.4
Very Small	19,624	4.0	43.7	7.7	5.4
Birth Interval					
1st	157,067	30.4	17.0	*6.3	*4.5
< 36	193,030	37.4	39.9	5.8	4.4
36+	165,780	32.1	34.5	5.6	3.5
Birth Order					
1	157,065	30.4	17.0	*6.3	*4.5
2	134,436	26.0	23.3	5.9	4.6
3	83,134	16.1	34.7	6.0	3.9
4	142,462	27.6	52.0	5.5	3.1
Have money for health care					
Not Problem	101,954	20.5	21.2	*7.0	*6.2
Problem	395,445	79.5	33.2	5.8	3.7
Has Health Insurance					
No	409,359	87.3	32.8	*6.1	*4.5
Yes	59,643	12.7	16.1	6.3	3.9
Community SES Quintiles					
1 (Highest)	117,186	20.2	9.6	*4.5	*4.2
2	101,302	20.0	17.8	4.8	4.2
3	103,795	20.1	28.9	5.0	3.9
4	100,611	20.0	42.6	6.0	4.2
5 (Lowest)	94,203	19.7	62.4	6.7	4.2
Total	532,680	100.0	31.1	*5.8	*4.2

*Significant at 0.05 in Mantel Haenszel test of homogeneity of the odds ratio

### Magnitude and variations in educational inequality in SAM

**Fig. 2:**
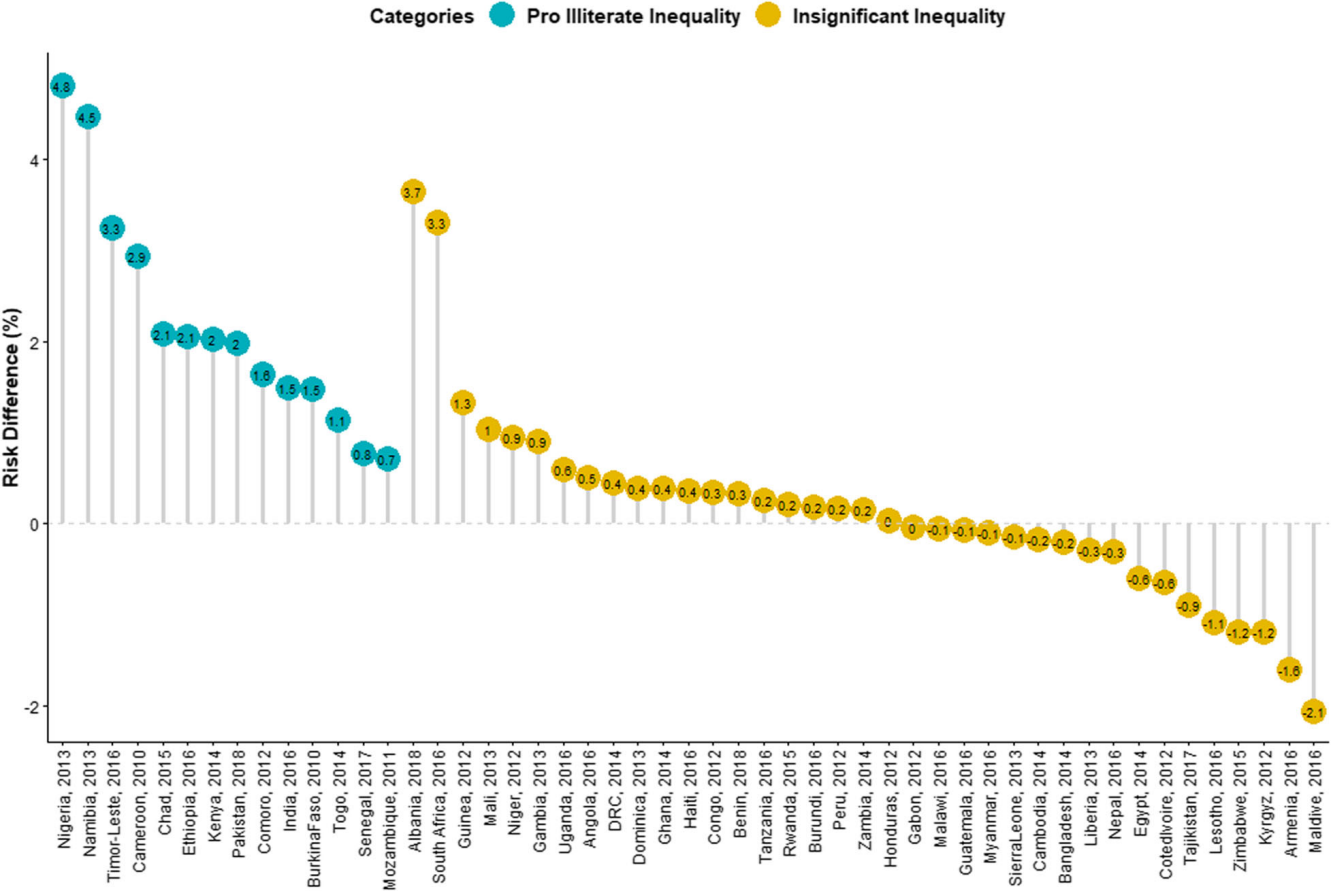
Risk difference between children born to uneducated and educated mothers in the prevalence of SAM by countries

Figures 1 and 2 show the RD between the children of uneducated and educated mothers across the 51 LMIC. Among the 51 countries, 14 countries had statistically significant pro-illiterate inequality (that is, prevalence of SAM is higher among children from uneducated mothers). None of the countries had statistically significant pro-literacy while 37 countries had no statistically significant inequality. As shown in Fig. 1, the educational difference was largest for Ethiopia (20.55 per 1000 children) and lowest for Malawi (− 0.50/1000) in the Eastern Africa. In Western Africa, the largest educational difference was in Nigeria (48.22/1000) and lowest for Cote d’Ivoire (− 6.41/1000). In the Caribbean, the difference was largest for Timor-Leste (32.60/1000) and lowest for Myanmar (− 0.96/1000). Burundi and Senegal with 2.5% weight each had the largest contribution to the pooled result. In the pooled analysis, Nigeria still had the highest pro-illiterate inequality (48.22/1000) and followed by Namibia (44.75/1000) as shown in Fig. 2. Overall, there was significant pro-illiterate in the total pooled sample of children in this study. The risk difference was 7.18 (95% Confidence Interval (CI): 3–12) per 1000 children among children of uneducated mothers compared with those of educated mothers as shown in the random effects in Fig. 1. The random effect shows the overall risk difference among all children born to educated and uneducated mothers irrespective of their countries. In Fig. 2, we used the colours blue, yellow and red to indicate statistically significant pro-illiterate inequality, no significant inequality and statistically significant pro-literate inequality respectively.

Two of the nine countries in Eastern Africa inequality, 2 of the countries in Middle Africa, none in Northern Africa, and only Namibia in Southern Africa showed statistically significant pro-illiteracy inequalities. Two of the 13 Western Africa countries and 2 of the five countries in Southern Asia showed statistically significant pro-illiterate inequality compared with only one country among the four countries studied in the Caribbean.

### Relationship between prevalence of SAM and magnitude of the educational inequality

**Fig. 3:**
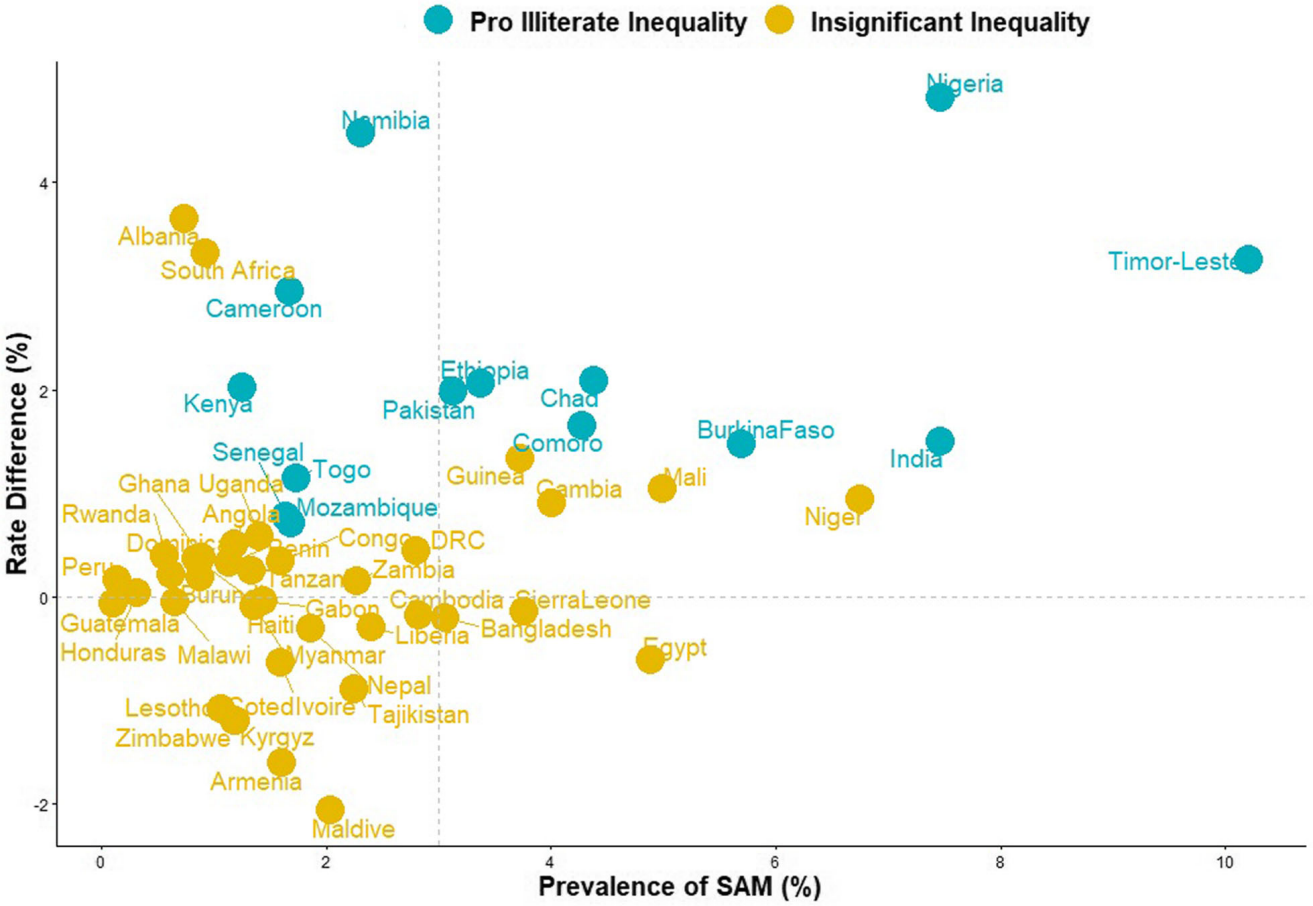
Scatter plot of rate of SAM and risk difference between children born to uneducated and educated mothers in LMIC

Figure 3 shows the level of relationship between the prevalence of SAM and the magnitude of the inequality across the 51 countries in this study. The 51 countries were categorized into 4: (1) High severe acute malnutrition and high pro-illiterate inequality countries such as Timor-Leste and Nigeria; (2) High severe acute malnutrition and high pro-literate inequality was not found in any country; (3) Low severe acute malnutrition and high pro-illiterate inequality in countries such as Namibia and Kenya; and (4) Low severe acute malnutrition and high pro-literate inequality was not found in any country. In Fig. 3, colours cyan, orange and red were used to depict statistically significant pro-illiterate inequality, no significant inequality and statistically significant pro-literate inequality respectively.

### Decomposition of educational inequality in the prevalence of SAM

**Fig. 4:**
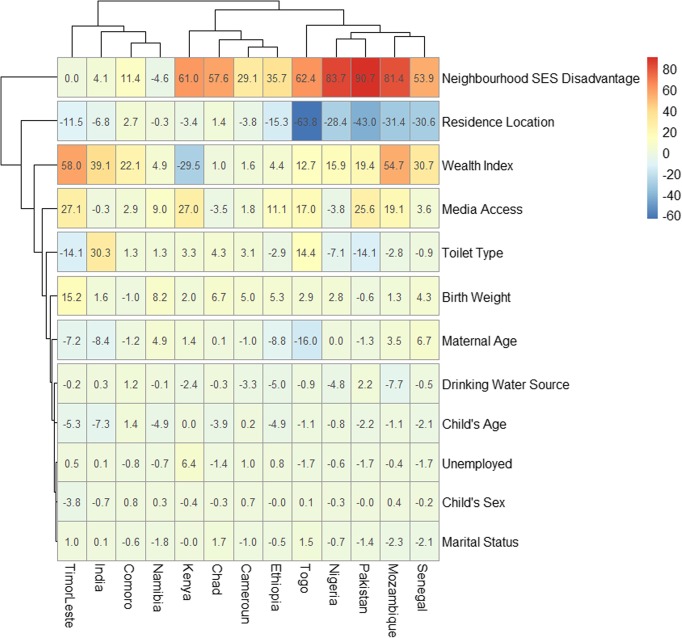
Contributions of differences in the distribution of the determinants of SAM to the total gap between children from uneducated and educated mothers by countries

Figure 4 shows a detail decomposition of the inequality due to compositional effects of the factors associated with SAM among the under-five children. There were variations in the important factors associated with the educational inequalities across the 51 countries. The “explained” (compositional component) and the “unexplained” (structural component) portions of the educational inequalities are depicted by red and blue colours respectively; the lighter the red colour the lower the percentage contribution of the “explained” portion and the lighter the blue colour, the lower the percentage contribution of the “unexplained” portion.

On the average, neighbourhood socioeconomic status disadvantage and, location of residence were the most significant factors in most of the countries studied. In Senegal, the highest contributions to the educational inequality in the prevalence of SAM was by neighbourhood socioeconomic disadvantage, followed by the location of residence, wealth index and access to media. Wealth index and media access narrowed the inequality in the development of SAM between children from educated and uneducated mothers. In Togo, location of residence was the highest contributor to the educational inequality followed by neighbourhood socioeconomic status disadvantage and then access to media. Marital status, child age and sex, birth weight and employment status of the mothers did not show any significant contribution to educational inequality in the development of SAM in any of the countries.

## Discussion

The goal of this study was to use the DHS data to decompose educational inequalities in the development of SAM across the 51 low- and middle-income countries. This study was carried out to improve the knowledge of the compositional and structural factors that are associated with educational inequalities in the development of SAM in the countries. The study is premised on the fact that SAM has continued to be a major public health challenge. The prevalence of SAM among children of illiterate and literate mothers varied significantly. We found significant educational-related differences that are better explained by structural and compositional factors which were nested both at the neighbourhood-level and the country-level. We also found wide inter-country differences viz-a-viz literacy level in the prevalence of SAM. The inter-country variations could be ascribed to the prevalent differences in individual country’s socioeconomic distribution, policies, strategies and existing level of intervention on child nutrition. Our findings are corroborated by some previous research which found similar differentials in the prevalence of SAM.

In particular, the analysis in this study shows the unequal distribution in the prevalence of SAM between the children of the educated and uneducated mothers. This suggests the presence of educational inequalities in the development of SAM among the children. In 13 of the 51 countries, pro-illiterate inequality SAM was significantly prevalent but pro-literate inequality, although higher in 16 countries, was insignificant in any of the countries. Among the countries which had significant pro-illiterate inequalities, risk difference used as the measure of inequality in our study showed that 8 to 48 per 1000 of children whose mothers were not educated will develop SAM compared with children from educated mothers.

Overall, there was significant pro-illiterate inequality among the total pooled sample of children in this study with 7 more children of every 1000 children of uneducated mothers developing SAM compared with children born to educated mothers. Educational attainment of caregivers is an important factor in whether a child develops SAM or not. Our finding aligns with previous studies which reported that children of uneducated mothers were associated with a poor range of nutritional outcomes such as stunting, wasting and malnutrition [7, 12, 17, 32–35]. This finding has several implications; first, there is a need for LMIC to develop child nutrition public health policies, interventions and programmes that particularly inform and train uneducated mothers on the need to provide their children with adequate nutrition.

Also, there is a need to increase the knowledge of mothers and households in general so that they can have a higher capacity to afford good nutrition for their children. Besides, governments may wish to subsidize children foods as a means of relieving a huge household burden of getting food for their wards. Nonetheless, such public health interventions should be all-encompassing. It should include health education and promotion, adequate communication, seminars, political will and the community and religious leaders’ participation. This is consistent with a UNICEF report that prevention and long term solutions to the burden of SAM will involve “dismantling unequal power structures, improving equitable access to health services and nutritious foods, promoting breastfeeding and optimal infant and young child feeding practices, improving water and sanitation, and planning for cyclic food shortages and emergencies” [4].

It is very evident from our analysis that compositional effects of the additional explanatory variables explored contributed to the majority of the inequalities in SAM between the children of the educated and the uneducated mothers in Chad, Timor-Leste and Mozambique. While in Togo, and Kenya, structural effects of the identified characteristics contributed mostly to the educational-inequalities in the development of SAM.

The decomposition analysis has shown that compositional factors including the neighbourhood SES, location of residence, wealth index and access to media were the greatest contributors to educational-related inequalities across the countries. Obviously, to attain a meaningful reduction in educational inequalities in SAM, there is a need to look outside the box and properly understand the connection among the structure, composition and context in which the children live. A wholesome approach should be used to address the challenges of educational inequalities in child health in general and in SAM in particular. This finding from our study underscores the advantage of enhancing both the compositional and structural characteristics if educational-related inequalities in SAM are to be reduced. Earlier reports on child malnutrition have clearly indicated the nuances of individual, community and country-level factors associated with child nutrition [2, 4, 8, 10].

We find interesting results in our attempt to map the relationships between the prevalence of SAM and educational inequality. Countries such as Namibia and Kenya had low prevalence of SAM and high pro-illiterate inequalities while countries such as Timor-Leste and Nigeria had a high prevalence of SAM and high pro-illiterate inequality. These variations can be explained by access to media, household wealth status, country-level policies and programmes for child nutrition, famine, war, internal displacement, political and economic instability. It is quite understandable that we did not find significant pro-literate inequality in any of the countries studied. An educated mother should engage in good nutritional practices for her wards.

Our findings on the effect of neighbourhood SES on the likelihood that children of an educated mother have SAM are in consonance with existing findings [36, 37]. These studies showed that the odds of better health outcomes are higher among residents in high socioeconomic areas than persons who reside in socioeconomically disadvantaged areas [36, 37]. It is therefore important that the countries with high SAM and high pro-illiterate inequalities in SAM rework their child nutrition policies by taking a cue from countries with a low SAM and low pro-literate inequalities. For instance, researchers and health programmers in such countries may wish to explore the differentials in child health and nutrition in Nigeria and Kenya. Why is SAM higher in Nigeria than in Kenya even though both countries have pro-illiteracy inequalities?

### Study limitations and strengths

We have used household wealth status as a proxy for household income as the DHS survey did not collect any information on household income. Hence, our findings may not be generalizable in countries where direct measurement of household income is available. While multilevel analysis has proved to be an efficient method for assessing disparities and to monitor health care indicators, Blinder-Oaxaca decomposition analysis is not an alternative measure of causality. It, however, gives robust evidence of inequalities after controlling for the exposure variables. There may be a need for further studies to examine the influence and association of structural and compositional factors with educational-inequalities in the prevalence of SAM. Nonetheless, our study has major strengths. As shown in Fig. 4, we quantified the magnitude of the explained and unexplained factors associated with our outcome measure. The study covered 51 LMIC using the DHS data is reputed for accuracy and comparability across countries.

## Conclusions

We identified that SAM is prevalent in most LMIC with wide educational variations. The occurrence of SAM was explained by the individual, household and community-level factor. The overall significance of our exposure variable in explaining the difference in SAM prevalence is a pointer that education of the whole population, especially the girl child who is a potential mother, is very important to child health. The advantages of education in human endeavour cannot be overemphasized. The low- and middle-income countries must improve their tactics in child nutrition with the goal of eradication of severe acute malnutrition which would eventually reduce child morbidity, opportunistic infections and mortality. To address the educational inequalities in SAM, an urgent child nutrition intervention is a must in the low- and middle- income countries, especially in those identified as having pro-illiterate inequalities as better education among all women will close the gaps and reduce the burden of SAM generally. We recommend further studies of other determinate causes of inequalities in severe acute malnutrition in low- and middle-income countries.

## Data Availability

The data supporting this article is available at http://dhsprogram.com. The data is publicly available but permission to use the data is required. The authors obtained permission from the data owners to use the data.

## Abbreviations

CI: Confidence Interval
DHS: Demographic and Health Survey
IRB: Institutional Review Board
LMIC: Low- And Middle-Income Countries
PSU: Primary Sample Unit
RD: Risk Difference
SAM: Severe Acute Malnutrition
SES: Socioeconomic Status
U5C: Under-Five Children
UNICEF: United Nations International Children’s Emergency Fund
WHO: World Health Organization
WHZ: Weight for Height Score
